# Diagnostic value of maternal alpha-fetoprotein variants in second-trimester biochemical screening for trisomy 21 and 18

**DOI:** 10.1038/s41598-022-16807-x

**Published:** 2022-08-10

**Authors:** Yiming Chen, Yijie Chen, Wenwen Ning, Wen Zhang, Liyao Li, Xiaoying Wang, Yixuan Yin, Huimin Zhang

**Affiliations:** 1 Department of Prenatal Diagnosis and Screening Center, Hangzhou Women’s Hospital (Hangzhou Maternity and Child Health Care Hospital), Teaching Hospital of Zhejiang Chinese Medical University, No. 369, Kunpeng Road, Shangcheng District, Hangzhou, 310008 Zhejiang China; 2 The Fourth School of Clinical Medicine of Zhejiang Chinese Medical University, Hangzhou, 310053 Zhejiang China

**Keywords:** Biochemistry, Developmental biology, Genetics, Diseases, Medical research

## Abstract

To evaluate the clinical predictive value of serum alpha-fetoprotein variants (AFP-L2, AFP-L3) in combination with maternal serum prenatal screening biomarkers in predicting fetal trisomy 21 and trisomy 18. We analyze the data of singleton pregnant women at 15–20^+6^ weeks of 731,922 gravidas from October 2007 to September 2019. The research objects were separated into the following groups: control (n = 569), trisomy 21 (n = 116), and trisomy 18 (n = 52). The cases were diagnosed by chromosomal karyotypic analysis of amniotic fluid cells. Level of AFP-L2 and AFP-L3 were detected in maternal serum among control women and patients. Receiver operator characteristic analysis, detection rate, false positive rate, false negative rate, positive predictive value, negative predictive value, positive likelihood ratio and negative likelihood ratio, comprehensive discriminant improvement, net weight classification improvement, decision curve analysis and Hosmer–lemeshow (H-L) test were used to investigate the predictive value of free β-hCG, AFP, AFP-L2 and AFP-L3 on the risk models of trisomy 21, 18. There was a statistically significant difference in maternal serum AFP-L2 and AFP-L3 multiple of the median (MoM) among the trisomy 21, trisomy 18, and control groups. The AUCs of AFP-L2 and AFP-L3 for the screening trisomy 21 and trisomy 18 fetus were 0.785, 0.758 and 0.775, 0.754. According to ROC, the optimal cut-off values of AFP-L2 and AFP-L3 for predicting trisomy 21 and trisomy 18 fetuses all were 1.09 MoM and 1.30 MoM, respectively. The risk-calculation model constructed by AFP-L2 + AFP-L3 MoM manifested better efficiency than the original single-value truncation method using AFP MoM alone. Compared with different modeling methods, the AUC of trisomy 21 fetuses predicted by AFP-L2 + AFP-L3 + free β-hCG achieved an optimal value (0.938), while the AUC of trisomy 18 fetus predicted by AFP-L2 + free β-hCG was the best (0.991). Compared with AFP, the IDI of AFP-L2 or AFP-L3 alone increased 9.56% and 12.34%; the NRI increased 26.50% and 26.70 in predicting trisomy 21. For trisomy 18, the IDI of AFP-L2 or AFP-L3 alone declined with 8.12% and 1.52%; the NRI declined with 13.84% and 8.54%. In the combined model, the model with best detection rate, false positive rate and positive likelihood ratio was AFP-L2 + AFP-L3 + free β-hCG, followed by AFP-L2 + free β-hCG and AFP-L3 + free β-hCG, and finally AFP + free β-hCG. Maternal serum AFP-L2 and AFP-L3 in the second trimester is a good marker for screening trisomy 21 and trisomy18 with high sensitivity and specificity. The combined screening results are better than the single marker, and the efficiency of AFP-L2 + AFP-L3 + free β-hCG is the best.

## Introduction

Down syndrome (DS) due to trisomy 21 is characterized by a wide range of physical and cognitive issues. It is generated by individuals having three copies of chromosome 21 rather than two, or copies of specific regions of chromosome 21^1^. Every pregnant woman should be actively screened for this chromosomal abnormality, as aneuploidy has a great impact on the health of the fetus with birth defects. Combined prenatal screening in early pregnancy, between 10 and 13^+6^ weeks of gestation, are powered to detect between 82 and 87% of DS cases, while in second trimester, at 15 to 22^+6^ weeks of gestation, detects 81% of DS cases^2,3^. Maternal serum screening is a simple, economical, and less-invasive method to predict the risk of fetal neural tube defects (NTDs), trisomy 21 and 18, by detecting maternal serum markers. The current screening for trisomy 21, 18 by applying markers such as maternal serum free human chorionic gonadotropin (free β-hCG) and alpha-fetoprotein (AFP) in second trimester has evolved into a routine obstetric examination^4^. Of 1,131,336 pregnant women who were evaluated at 56 laboratory screenings in the United States, 36% were screened by independent early-pregnancy tests, 48% by independent middle-pregnancy tests, and 16% by integrated-screening results in 2020^5,6^. Although non-invasive prenatal testing (NIPT) technology has recently become available and is now extensively applied to prenatal screening^7^, there remains a very broad appeal for screening in the second trimester. However, NIPT cannot be used for screening open NTDs, and it is not widely promoted because of its high cost.

Although AFP is one of the principal markers for trisomies 21 and 18 in second-trimester serum screening that has been routinely carried out in various geographical regions, its sensitivity remains low. After integrating with lens culinaris agglutinin (LCA), serum AFP can be dissociated into three bands by electrophoresis: LCA-non-bound (AFP-L1 and AFP-L2) and LCA-bound AFPs (AFP-L3)^8–10^.

AFP-L3 which is considered as a new generation of tumor marker is usually referred to as AFP heteroplasmy and binds to lentinan^11–13^. Van Staden et al. also showed that the rank correlation analysis of AFP L2 and L3 bands in combination distinguish patients with hepatocellular carcinoma from those with other liver diseases (*P* < 0.05)^14^.

The production of AFP in the fetal liver and yolk sac appears to be decreased in DS. The fetus products only a small amount to the AFP reserve of non-concanavalin A -reactive amniotic fluid, such that the AFP comes almost entirely from the yolk sac. Although AFP, primarily synthesized in the yolk sac, ceases after the termination of the first trimester of pregnancy, the pool is maintained until the third trimester of pregnancy, indicating that the protein in the amniotic fluid disappears very slowly^15^. Chen et al.^9^ showed that three possible electrophoretic bands for AFP were L1, L2, and L3, and that yolk sac tumors generated darker L2 and lighter L3 bands, and that hepatocellular carcinoma produced significant L1 and lighter L3 bands.

Compared with single marker, the combination of AFP-L3 and AFP could improves diagnostic performance for hepatocellular carcinoma^16^. The results showed that the efficiency of AFP-L3 in screening fetal trisomy 21 was better than that of AFP^17^. In our preliminary study, we evaluated the effectiveness of AFP-L2 and AFP-L3 screening for fetal trisomy 18 in the second trimester of pregnancy (n = 39), and we demonstrated that AFP-L2 and AFP-L3 could improve sensitivity and specificity^18^. A previous retrospective case–control study also showed that superior sensitivity and specificity for fetal DS screening in applied with AFP-L2 and AFP-L3 risk models during the second trimester^19^. The combination of these detection methods comprises comprehensive or serum-comprehensive, gradual-sequential and/or sequential screening, and it improves the detection rate (DR) compared with single-detection methods.

Based on our previous experiments^19^, we herein increased the number of control and case groups and adopted a retrospective case–control study method. We separated our subjects into a control group and case group, to investigate the relationship and predictive value of serum AFP-L2 and AFP-L3 levels in pregnant women with prenatal diagnosis of trisomy 21 and 18.

## Material and methods

### Study population

We retrospectively investigated second-trimester pregnant women who underwent prenatal screening at Hangzhou Women’s Hospital (Hangzhou Maternity and Child Health Care Hospital), and Maternal and Child Health Hospital of Yuhang District, Hangzhou, China, from October 2007 to September 2019, and we implemented a case–control analysis of 731,922 gravidas at 15–20^+6^ weeks of gestation. The subjects were divided into trisomy 18 group (n = 52), trisomy 21 group (n = 116), and control group (n = 569)^20^. Amniocentesis and karyotypic analysis of amniotic fluid cells were underwent for invasive prenatal diagnosis in the case groups. The study was approved by Hangzhou Women’s Hospital (Hangzhou Maternity and Child Health Care Hospital) ethics committee, and the approval number was [2021] medical ethics A (3)—02. The informed consent was waived by Hangzhou Women’s Hospital (Hangzhou Maternity and Child Health Care Hospital) ethics committee due a retrospective nature of the study.

These cases were diagnosed according to the criteria established by Chinese Birth Defect Monitoring Network and International Classification of Diseases (ICD-10)^21,22^. The exclusion criteria were: (1) women undergoing multiple pregnancy, (2) patients with severe medical conditions and other pregnancy complications, (3) habitual smokers, (4) infants conceived from assisted reproductive technology, (5) patients in whom follow-up results showed NTDs or other serious birth defects, (6) patients with incomplete individual information, and (7) patients whose pregnancy information did not match its cognate serum sample.

### Reagents and instruments

AFP-L2 and AFP-L3 reagent (BIM, San Francisco, CA, USA) using a double-antibody, one-step enzyme-linked immunosorbent assay (ELISA), RT-6100 microplate reader (Rayto, Shenzhen, China), 988 plate washing machine (Tianshi, Beijing, China), and 1235 automatic time-resolved fluorescence immunoassay analyzer (DELFIA, Perkin Elmer, USA) were implemented, with PAPP-A and free β-hCG matching kit.

### Second-trimester screening

2–3 mL fasting venous blood of pregnant women was collected for the detection of prenatal screening markers. The blood samples were centrifuged at 2500 r/min for 5 min approximately 30 min after collection, and the remaining specimens were stored in a – 80 °C refrigerator within 1 week of the initial examination. Before measurement, serum samples and corresponding data were matched with the case group and the control group. The DELFIA method was used to measure the levels of pregnancy-associated plasma protein A (PAPP-A) and free β-hCG levels in maternal serum. We then measured the levels of AFP-L2 and AFP-L3 by ELISA^20,23^.

### Calculation of multiples of the median (MoMs) and establishment of risk models

Maternal aneuploidy screening markers varied with maternal weight and gestational age (GA) in unaffected pregnancies. AFP, free β-hCG, AFP-L2, and AFP-L3 concentrations were represented as MoMs to reduce the deviation caused by GA and maternal weight^20,24–26^.

1 $$MoM = \frac{Original\;Conj.}{{Median}}$$

2 $$Adjusted\_MoM = \frac{MoM}{{GA\_Med \times Weight\_Med}}$$

The formula for maternal-age risk values was as follows. Age represented the maternal age and risk _age_ was risk value for maternal age^24,26^:

3 $${risk}_{age}=0.0000697+{exp \, }^{-18.4367+0.286*(age-0.5)}.$$

Likelihood ratio calculation^25,27^:

4 $$LR_{multinorm} = \frac{{{\text{Likelihood}}\;{\text{of Trisomy }}21\;{\text{ or}} \; {\text{Trisony}} \; 18}}{{\text{Likelihood of controls}}},$$

5 $$risk_{T21,\;T18} = \frac{1}{{LR_{multinorm} \times risk_{{\text{maternal age}}} }}.$$

### The models below were constructed using the above steps

The single-index models comprised:

Model A: free β-hCG; Model B: AFP; Model C: AFP-L2; Model D: AFP-L3;

The dual-index models comprised:

Model E: AFP + free β-hCG; Model F: AFP-L2 + free β-hCG; Model G: AFP-L3 + free β-hCG; Model H: AFP-L2 + AFP-L3.

The triple-index model comprised:

Model I: free β-hCG + AFP-L2 + AFP-L3.

### Statistical analysis

SPSS version 21.0 (IBM, Armonk, NY, USA) software was used for data statistics. We exploited the one-sample Kolmogorov–Smirnov test for skewed data and the median and percentile (M [P_2.5_, P_97.5_]) to determine normality of the data. We compared two or more groups using the Mann–Whitney U test or Kruskal–Wallis H test, respectively. We use the DR, positive predictive value, negative predictive value, positive likelihood ratio (+LR) and negative likelihood ratio (−LR) values to assess model performance. Integrated discrimination improvement (IDI), net reclassification improvement indicators (NRI), and decision-curve analysis (DCA) were used to evaluate the T21 and T18 risk models performance. The values of area under the curve (AUC) were determined using receiver operator characteristics (ROC) curves to assess the diagnostic value for trisomy 21 and 18^28^. The optimum cut-off, AUC, sensitivity and specificity were determined by the maximum Yoden index. Hosmer–lemeshow (H-L) test was used to evaluate the calibration degree of the model. H-L test *P* > 0. 05 indicated that the predicted value of the model fitted well with the actual observed value and the model calibration degree was good^29^. It was generally considered that AUC ranged from 0. 70 to 0. 80 was medium and AUC > 0.80 was high. *P* < 0.05 denoted statistically different differences.

### Supplement

All methods performed in this article were in accordance with the relevant guidelines and regulations. For research involving human participants, we have identified the committee that approved the research, confirmed that all research was performed in accordance with relevant guidelines.

### Ethics approval

The study was approved by Hangzhou Women’s Hospital (Hangzhou Maternity and Child Health Care Hospital) ethics committee, and the approval number was [2021] medical ethics A (3)—02. The informed consent was waived by Hangzhou Women’s Hospital (Hangzhou Maternity and Child Health Care Hospital) ethics committee due a retrospective nature of the study.

## Results

### Comparison of basic data

Although we observed no significant difference in pregnancy weight between the case and control groups, maternal age in the control group was significantly lower than that in the trisomy 21 group (*P* = 0.004). Maternal age in the trisomy 18 group showed a tendency to be lower than that in the control group, but there is no statistically significant difference (*P* = 0.810, Table 1).

**Table 1:** Comparison of basic data in three groups.

Group	Control (n = 569)^20^	Trisomy 21 (n = 116)	Trisomy 18 (n = 52)	*P* (Trisomy 21)	*P *(Trisomy 18)
Maternal age (years)	28.59 (21.93–36.00)	30.00 (23.00–41.25)	28.00 (22.24–41.00)	0.004**	0.810
Maternal weight (kg)	54.00 (43.48–73.00)	53.30 (44.91–74.25)	56.00 (40.33–81.46)	0.559	0.089
Gestational age (days)	120.00 (106.00–140.00)	119.00 (109.00–136.00)	118.00 (105.00–131.00)	0.022**	0.022**

Data are presented as median (P_2.5_–P_97.5_). **P < 0.05.

### Comparison of serum AFP-L2, AFP-L3, free β-hCG, and AFP levels among the case and control groups

**Figure 1:**
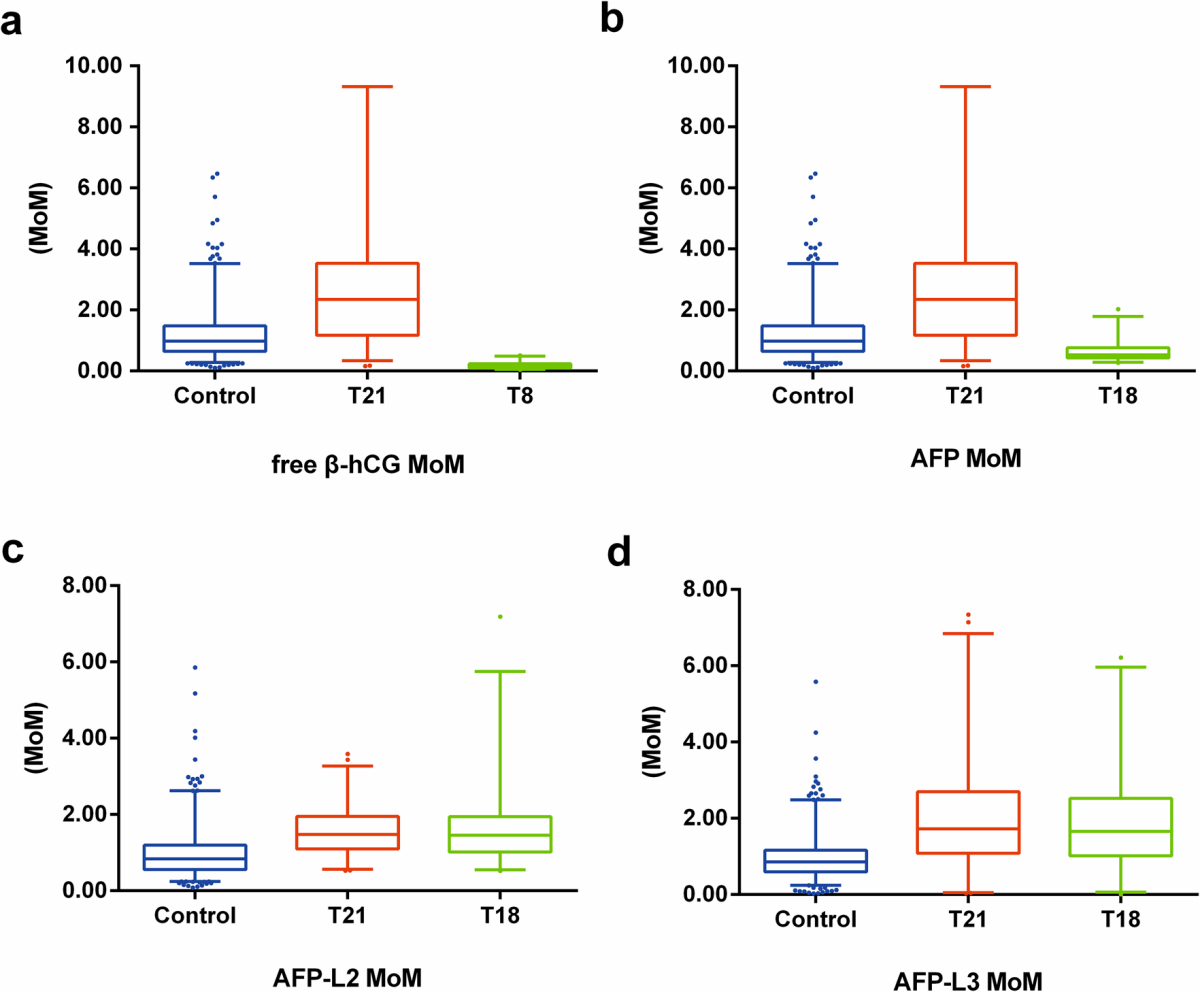
Comparison of MoMs for serum-free β-hCG, AFP, AFP-L2, and AFP-L3 in the two groups. (**a**) free β-hCG MoM of Group; (**b**) AFP MoM of Group; (**c**) AFP-L2 MoM of Group; (**d**) AFP-L3 MoM of Group. AFP, alpha-fetoprotein; free β-hCG, free β-subunit of human chorionic gonadotropin; AFP-L2, alpha-fetoprotein variant L2; AFP-L3, alpha-fetoprotein variant L3; MoM, multiple of the median; T21, trisomy 21; T18, trisomy 18.

The AFP-L2 levels of gravidas with fetal trisomy 21 and trisomy 18 were 1.48 (0.56–3.27) and 1.46 (0.55–5.75) MoMs, which were respectively higher than the 0.83 (0.24–2.62)^20^ in the control subjects (all *P* < 0.001). The levels of AFP-L3 in gravidas carrying trisomy 21 and trisomy 18 fetuses were 1.73 (0.05–6.84) and 1.66 (0.60–5.96) MoMs, respectively, which were also higher than the 0.85 (0.24–2.48) MoM^20^ in the control subjects (all *P* < 0.001). However, the AFP levels of pregnant women with trisomy 21 and trisomy 18 fetuses were lower than in the control subjects (all *P* < 0.001). The free β-hCG of the trisomy 21 group was elevated relative to the control subjects, and that of the trisomy 18 group was inferior to that of the control subjects (all *P* < 0.001, Table 2 and Fig. 1).

**Table 2:** Comparison of Maternal serum screening indicators in three groups.

Group (MoM)	Control (n = 569)^20^	Trisomy 21 (n = 116)	Trisomy 18 (n = 52)	*P* (Trisomy 21)	*P* (Trisomy 18)
AFP	1.01 (0.54–1.86)	0.87 (0.43–2.37)	0.53 (0.28–1.79)	< 0.001*	< 0.001*
free β-hCG	0.98 (0.28–3.52)	2.34 (0.34–9.32)	0.16 (0.47–0.49)	< 0.001*	< 0.001*
AFP-L2	0.83 (0.24–2.62)	1.48 (0.56–3.27)	1.46 (0.55–5.75)	< 0.001*	< 0.001*
AFP-L3	0.85 (0.24–2.48)	1.73 (0.05–6.84)	1.66 (0.60–5.96)	< 0.001*	< 0.001*

AFP, alpha-fetoprotein; free β-hCG, free beta subunit of human chorionic gonadotropin; AFP-L2, α-fetoprotein heterogeneity L2; AFP-L3, α-fetoprotein heterogeneity L3; MoM, multiple of median. Data are presented as median (P_2.5_–P_97.5_). *Statistically significant difference. *P < 0.001.

### The screening value of individual- and combined-index for trisomy 21, 18

**Figure 2:**
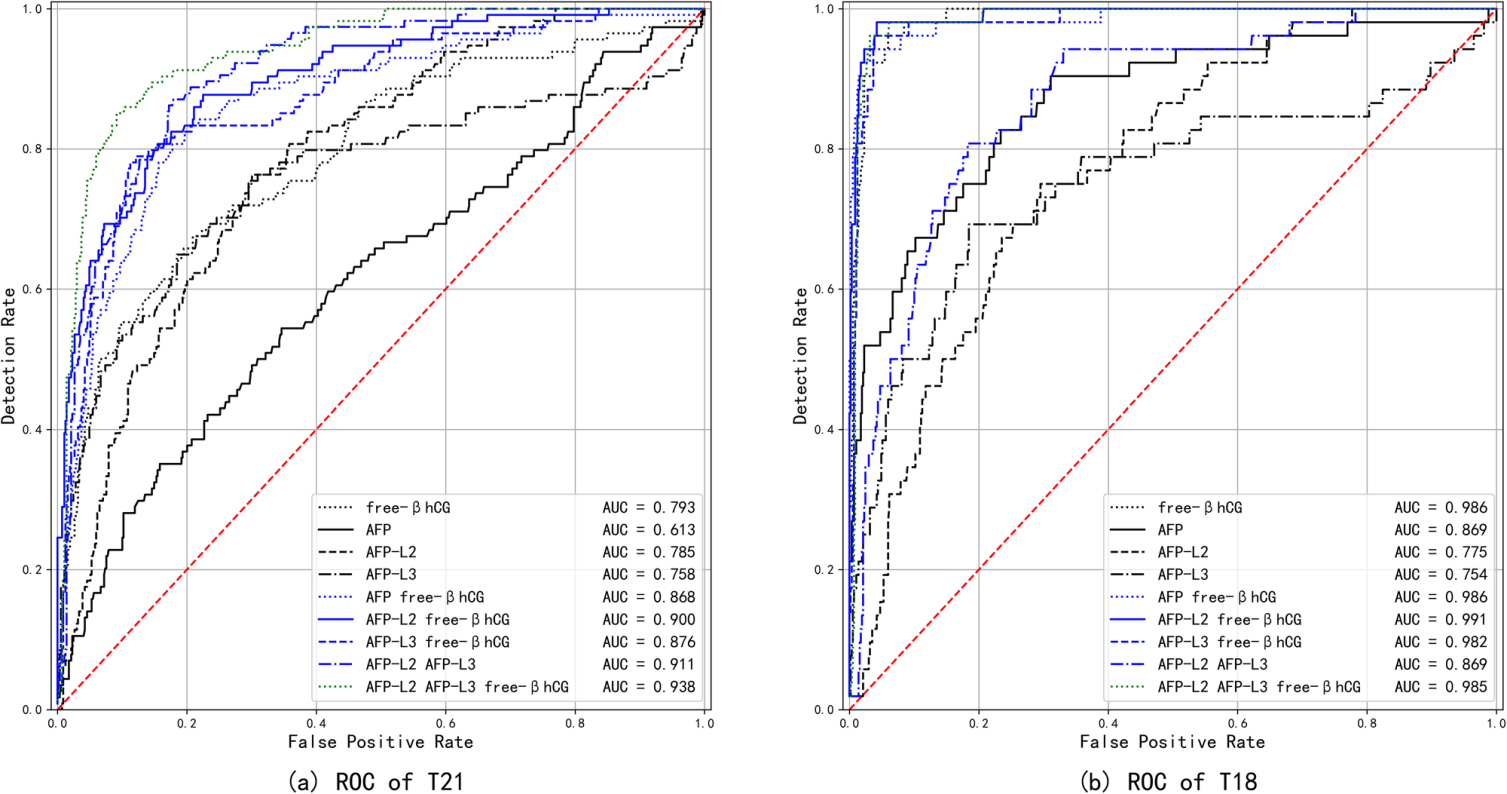
(**a**) ROC curves of trisomy 21 as predicted by different models. AFP, alpha-fetoprotein; free β-hCG, free β-subunit of human chorionic gonadotropin; AFP-L2, alpha-fetoprotein variant L2; AFP-L3, alpha-fetoprotein variant L3; MoM, multiple of the median; ROC, receiver-operating characteristic. (**b**) ROC curves of trisomy 18 as predicted by different models. AFP, alpha-fetoprotein; free β-hCG, free β-subunit of human chorionic gonadotropin; AFP-L2, alpha-fetoprotein variant L2; AFP-L3, alpha-fetoprotein variant L3; MoM, multiple of the median; ROC, receiver-operating characteristic.

The AUCs for AFP-L2 in the screening of trisomy 21, 18 fetuses were higher than those for AFP in trisomy 21, and lower than those for trisomy 18 (0.785 and 0.758 vs, 0.613; 0.775 and 0.754 vs, 0.869, respectively). In addition, the optimal threshold values for AFP-L2 and AFP-L3 in predicting T21 and T18 in fetuses all were 1.09 and 1.30 MoMs, respectively. The AUCs for free β-hCG in trisomy 21 and 18 fetuses were 0.793 and 0.986, respectively, higher than for AFP-L2, AFP-L3, or AFP (as shown in Table 3). The risk-calculation model constructed with AFP-L2 + AFP-L3 MoM exhibited better screening efficiency than the original single-value truncation method using AFP MoM. Compared with different modeling methods, the AUC for trisomy 21 fetuses as predicted by Model I was the highest (AUC = 0.938, Fig. 2a), while for trisomy 18, Model F achieved the optimal AUC (AUC = 0.991, Fig. 2b).

**Table 3:** The value of individual and combined index screening for the Trisomy 21 and Trisomy18.

Screening method	Youden	Sensitivity	Specificity	Cut-off	AUC	95%CI	*P*	DR	PPV	NPV	FPR	FNR	+LR	−LR
**Trisomy 21**														
Free β-hCG	0.466	0.675	0.791	1.61	0.793	0.744–0.842	< 0.001*	0.772	0.393	0.924	0.209	0.325	3.230	0.410
AFP	0.198	0.544	0.654	0.90	0.613	0.553–0.673	< 0.001*	0.635	0.239	0.877	0.346	0.456	1.571	0.698
AFP-L2	0.463	0.763	0.699	1.09	0.785	0.742–0.827	< 0.001*	0.710	0.337	0.936	0.301	0.237	2.539	0.339
AFP-L3	0.465	0.649	0.815	1.30	0.758	0.699–0.818	< 0.001*	0.788	0.413	0.921	0.185	0.351	3.518	0.430
AFP + free β-hCG	0.630	0.807	0.822	1/790	0.868	0.830–0.906	< 0.001*	0.820	0.477	0.955	0.178	0.193	4.546	0.235
AFP-L2 + free β-hCG	0.652	0.877	0.775	1/1412	0.900	0.868–0.932	< 0.001*	0.792	0.439	0.969	0.225	0.123	3.899	0.158
AFP-L3 + free β-hCG	0.668	0.781	0.888	1/690	0.876	0.839–0.913	< 0.001*	0.870	0.582	0.953	0.112	0.219	6.941	0.247
AFP-L2 + AFP-L3	0.691	0.871	0.821	1/1398	0.911	0.885–0.937	< 0.001*	0.829	0.498	0.969	0.179	0.129	4.857	0.158
AFP-L2 + AFP-L3 + free β-hCG	0.759	0.851	0.909	1/850	0.938	0.917–0.960	< 0.001*	0.899	0.651	0.968	0.091	0.149	9.311	0.164
**Trisomy 18**														
free β-hCG	0.902	0.962	0.940	0.37	0.986	0.977–0.995	< 0.001*	0.942	0.595	0.996	0.060	0.038	16.092	0.041
AFP	0.593	0.827	0.766	0.80	0.869	0.811–0.926	< 0.001*	0.771	0.244	0.980	0.234	0.173	3.538	0.226
AFP-L2	0.455	0.750	0.705	1.09	0.775	0.716–0.834	< 0.001*	0.709	0.188	0.969	0.295	0.250	2.540	0.355
AFP-L3	0.508	0.692	0.815	1.30	0.754	0.667–0.840	< 0.001*	0.805	0.255	0.967	0.185	0.308	3.752	0.377
AFP + free β-hCG	0.911	0.942	0.968	1/521	0.986	0.970–1.000	< 0.001*	0.966	0.731	0.995	0.032	0.058	29.787	0.060
AFP-L2 + free β-hCG	0.939	0.981	0.958	1/1034	0.991	0.982–0.999	< 0.001*	0.960	0.680	0.998	0.042	0.019	23.252	0.020
AFP-L3 + free β-hCG	0.923	0.962	0.961	1/671	0.982	0.968–0.996	< 0.001*	0.961	0.694	0.996	0.039	0.038	24.869	0.040
AFP-L2 + AFP-L3	0.625	0.808	0.817	1/1482	0.869	0.821–0.917	< 0.001*	0.816	0.288	0.979	0.183	0.192	4.419	0.235
AFP-L2 + AFP-L3 + free β-hCG	0.930	0.962	0.968	1/560	0.985	0.974–0.995	< 0.001*	0.968	0.735	0.996	0.032	0.038	30.395	0.040

AFP, alpha-fetoprotein; free β-hCG, free β subunit of human chorionic gonadotropin; AFP-L2, α-fetoprotein heterogeneity L2; AFP-L3, α-fetoprotein heterogeneity L3; MoM, multiple of median; DR, Detection rate; FPR, False positive rate; FNR, False negative rate; PPV, Positive predictive value; NPV, Negative predictive value; +LR: Positive likelihood ratio; −LR: Negative likelihood ratio. *P < 0.001.

### The evaluation of risk models

The AUCs of the single-index model for predicting trisomy 21 in decreasing order were AFP-L2 > AFP-L3 > AFP, and the AUCs of the single-index model for predicting trisomy 18 were AFP > AFP-L2 > AFP-L3. In the multi-index combination model, the ranking of DR, false-positive rate (FPR), and +LR evaluation indices were Model I > Model F > Model G > Model E, Table 3.

The replacement of AFP with AFP-L2 or AFP-L3 increased the IDI by 9.56% and 12.34%, respectively, and with NRI by 26.50% and 26.70, respectively, in predicting trisomy 21.

The replacement of free β-hCG by AFP-L2 or AFP-L3 combined with free β-hCG increased the IDI by 7.86% and 12.15%, respectively; and NRI by 15.61% and 17.21%, respectively. The IDI and NRI for T21 as predicted by Model I instead of Model H were 5.22% and 13.53%, respectively (Table 4).

**Table 4:** NRI and IDI for assessing improvement in model performance after adding AFP-L2, AFP-L3 to predicting Trisomy 21,18.

New method	Old method	IDI (%)	*P* value for IDI	NRI (%)	*P* value for NRI
**Trisomy 21**					
AFP-L2	AFP	9.56	< 0.001*	26.50	< 0.001*
AFP-L3	AFP	12.34	< 0.001*	26.70	< 0.001*
AFP-L2 + AFP-L3	AFP	8.12	< 0.001*	40.90	< 0.001*
AFP + free β-hCG	AFP	14.88	< 0.001*	37.40	< 0.001*
AFP + free β-hCG	free β-hCG	9.51	< 0.001*	10.54	0.008 **
AFP-L2 + free β-hCG	free β-hCG	7.86	< 0.001*	15.61	< 0.001*
AFP-L3 + free β-hCG	free β-hCG	12.15	< 0.001*	17.21	< 0.001*
AFP-L2 + AFP-L3 + free β-hCG	free β-hCG	7.97	< 0.001*	27.57	< 0.001*
AFP-L2 + free β-hCG	AFP-L2	3.67	0.003**	15.98	0.002 **
AFP-L2 + AFP-L3	AFP-L2	− 1.44	0.807	14.40	0.006 **
AFP-L2 + AFP-L3 + free β-hCG	AFP-L2	3.78	0.012**	27.93	< 0.001*
AFP-L3 + free β-hCG	AFP-L3	5.18	0.005**	17.38	0.002 **
AFP-L2 + AFP-L3	AFP-L3	− 4.22	0.978	14.21	0.005 **
AFP-L2 + AFP-L3 + free β-hCG	AFP-L3	1.00	0.319	27.74	< 0.001*
AFP-L2 + AFP-L3 + free β-hCG	AFP-L2 + free β-hCG	0.11	0.458	11.95	0.003**
AFP-L2 + AFP-L3 + free β-hCG	AFP-L3 + free β-hCG	− 4.18	1.000	10.35	0.002**
AFP-L2 + AFP-L3 + free β-hCG	AFP-L2 + AFP-L3	5.22	< 0.001*	13.53	0.002**
**Trisomy 18**					
AFP-L2	AFP	− 8.12	0.998	− 13.84	0.947
AFP-L3	AFP	− 1.52	0.668	− 8.54	0.815
AFP-L2 AFP-L3	AFP	− 12.40	1.000	− 2.26	0.610
AFP + free β-hCG	AFP	17.35	< 0.001*	31.05	< 0.001*
AFP + free β-hCG	free β-hCG	18.55	< 0.001*	0.19	0.478
AFP-L2 + free β-hCG	free β-hCG	17.05	< 0.001*	2.10	0.150
AFP-L3 + free β-hCG	free β-hCG	9.81	< 0.001*	− 1.57	0.786
AFP-L2 + AFP-L3 + free β-hCG	free β-hCG	− 0.75	0.707	0.71	0.366
AFP-L2 + free β-hCG	AFP-L2	23.97	< 0.001*	46.80	< 0.001*
AFP-L2 + AFP-L3	AFP-L2	− 4.28	0.954	11.58	0.072
AFP-L2 + AFP-L3 + free β-hCG	AFP-L2	6.17	0.005**	45.42	< 0.001*
AFP-L3 + free β-hCG	AFP-L3	10.12	0.001**	37.83	< 0.001*
AFP-L2 + AFP-L3	AFP-L3	− 10.88	0.999	6.28	0.220
AFP-L2 + AFP-L3 + free β-hCG	AFP-L3	− 0.44	0.551	40.11	< 0.001*
AFP-L2 + AFP-L3 + free β-hCG	AFP-L2 + free β-hCG	− 17.81	1.000	− 1.39	0.688
AFP-L2 + AFP-L3 + free β-hCG	AFP-L3 + free β-hCG	− 10.56	1.000	2.28	0.001**
AFP-L2 + AFP-L3 + free β-hCG	AFP-L2 + AFP-L3	10.44	< 0.001*	33.84	< 0.001*

AFP, alpha-fetoprotein; free β-hCG, free beta subunit of human chorionic gonadotropin; AFP-L2, α-fetoprotein heterogeneity L2; AFP-L3, α-fetoprotein heterogeneity L3; IDI, Integrated Discrimination Improvement; NRI, Net Reclassification Improvement. *P < 0.001, **P < 0.05.

The replacement of AFP by AFP-L2 or AFP-L3 decreased the IDI by -8.12% and -1.52%, respectively, and NRI by − 13.84% and − 8.54%, respectively, in predicting trisomy 18. The replacement of free β-hCG by AFP-L2 or AFP-L3 combined with free β-hCG increased the IDI by 17.05% and 2.10%, respectively; and NRI by 9.81% and − 1.57%, respectively. The IDI and NRI for T21 as predicted by Model I instead of Model H were 10.44% and 33.84%, respectively.

**Figure 3:**
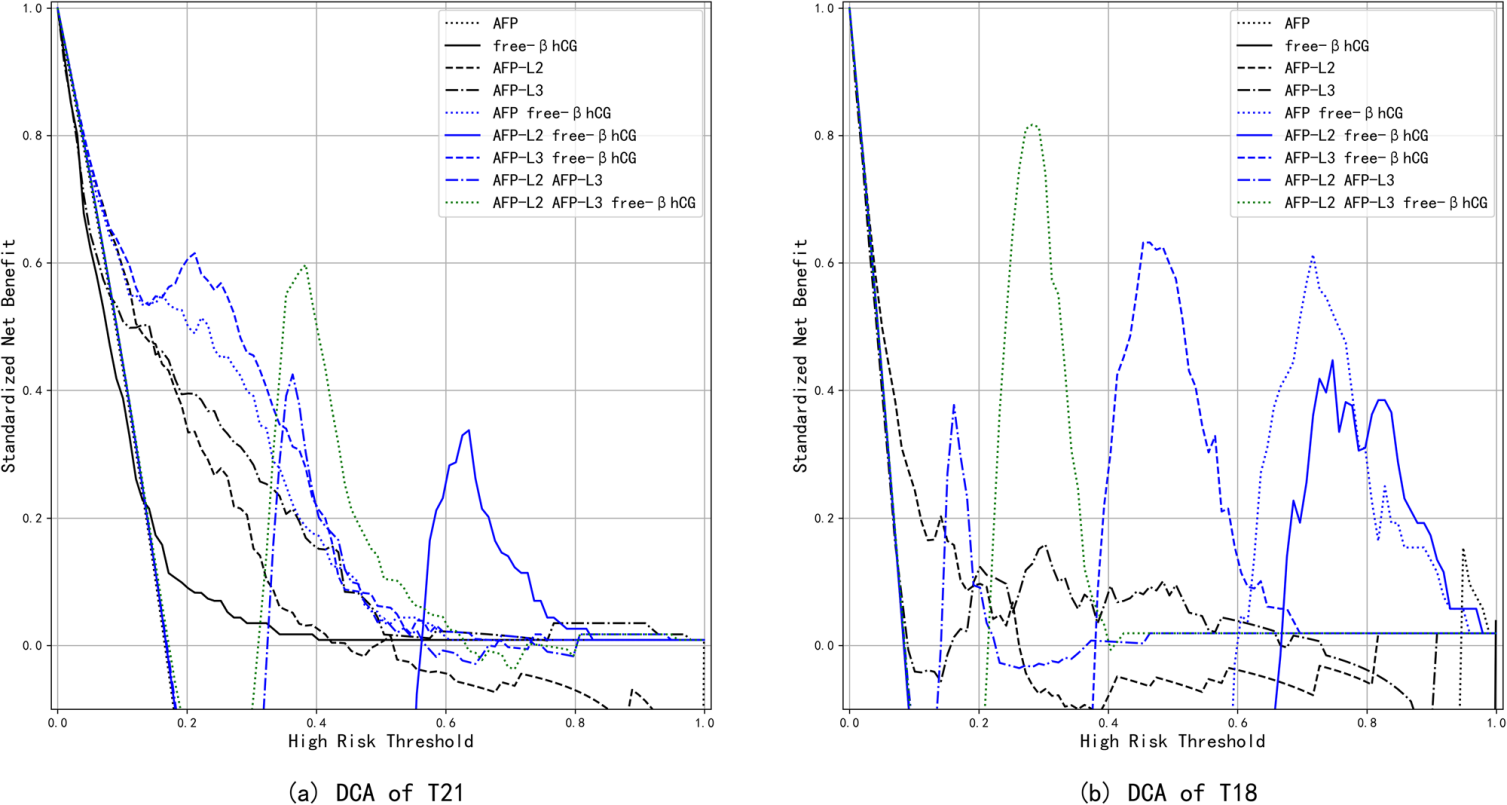
(**a**) DCAs of trisomy 21 as predicted by different models. AFP, alpha-fetoprotein; free β-hCG, free β-subunit of human chorionic gonadotropin; AFP-L2, alpha-fetoprotein variant L2; AFP-L3, alpha-fetoprotein variant L3; T21, trisomy 21; DCA, decision-curve analysis. (**b**) DCAs of trisomy 18 as predicted by different models. AFP, alpha-fetoprotein; free β-hCG, free β-subunit of human chorionic gonadotropin; AFP-L2, alpha-fetoprotein variant L2; AFP-L3, alpha-fetoprotein variant L3; T18, trisomy 18; DCA, decision-curve analysis.

When the risk threshold was < 0.40, the decision curve analyses for predicting T21 using different models were ranked Model H > Model F > Model E > Model D > Model C > Model B > Model A > Model G > Model I (Fig. 3a). There were different models that comprised DCA predictive ability for trisomy 18, and the top models were Model B, Model E, Model I, and Model D (Fig. 3b).

H-L test for trisomy 21, there was no statistical difference between AFP-L2 + AFP-L3 and AFP-L2 + free β-hCG (Z = 0.402, *P* > 0.05) and no significant difference between AFP-L2 and AFP-L3 (Z = 0.762, *P* > 0.05); there was statistical difference between other models for trisomy 21 H-L test (all *P* < 0.05). For Trisomy 18, there was no statistical difference between AFP and AFP-L2 + AFP-L3; AFP-L2 and AFP-L3; free β-hCG + AFP-L2 + AFP-L3 + free β-hCG (all *P* > 0.05), but there were significant differences among other models in the H-L test of trisomy 18 (all *P* < 0.05).

## Discussion

Trisomy 21 and trisomy 18 are the most common chromosomal abnormalities observed in neonatal birth defects. After birth, children with these anomalies cannot provide for themselves, and this engenders serious economic and social demands from families and society. Therefore, early prenatal diagnosis and intervention with certain measures are particularly important. The use of highly sensitive and specific markers for prenatal screening in early and middle pregnancy has become a recent focus in prenatal diagnosis and screening research. Previous studies have revealed a low sensitivity of maternal serum AFP screening for Trisomy 21 and 18 fetuses in second-trimester^30^. In the present study, we evaluated the effect of combining free β-hCG after replacing AFP with AFP-L2 or AFP-L3 on predicting trisomies 21 and 18.

The research showed that the AFP-L2 levels of gravidas screening for trisomy 21 and 18 fetuses in second trimester were higher than in the control subjects (1.48 and 1.46 vs. 0.83 MoM, respectively) (*P* < 0.001). Yu et al.^17^ also showed that the average level of serum AFP-L2 with trisomy 21 fetuses was higher than that of normal subjects. The consequences of our research also revealed that the AFP-L3 levels of gravidas carrying trisomy 21, 18 fetuses were augmented relative to those in the control subjects (1.73 MoM, 1.66 MoM vs. 0.85 MoM, respectively) (all *P* < 0.001). Similarly, Feng et al.^31^ demonstrated that the ratio of serum AFP-L3 to AFP in gravidas with a DS fetus was higher than that in pregnant women carrying a healthy fetus. However, Huai et al.^32^ showed that the level of serum AFP-L3 MoM in the normal control subjects was significantly higher than that in gravidas with DS fetuses (*P* < 0.05); this diverged from the results of our study, and it may be related to the different detection methods used to determine AFP-L3. However, the preliminary results of the present study revealed that maternal serum AFP-L2 and AFP-L3 levels of the control subjects were significantly lower than those of DS fetuses (all *P* < 0.01)^19^. Similarly, Wu et al.^33^ demonstrated that the concentrations of AFP-L2 and AFP-L3 in women with healthy fetuses were lower than that of DS fetuses, which was resemble to the data from the current study.

In the current research, we determined the levels of AFP-L2 and AFP-L3 by ELISA, and we showed that the AUC screening for trisomy 21, 18 fetuses using AFP-L2 was higher than that with AFP for trisomy 21, and lower than that for trisomy 18 (0.785 and 0.775 vs. 0.613 and 0.869, respectively). The AUCs screening for trisomy 21, 18 fetuses with AFP-L3 were 0.758 and 0.754, respectively, which were higher than AUCs using AFP (0.613 and 0.869). These results showed that the diagnostic values of AFP-L2 and AFP-L3 for trisomy 21, 18 were better than that of AFP. After denaturation of AFP, it can be easily refolded into both forms of recombinantly produced AFP under the conditions of dilution and redox reaction, and it is detectable by ELISA^34^.

Compared with the study by Long, our AUC for AFP-L3 screening of trisomy 21 in our study was higher than the ratio of AFP-L3 to AFP (0.758 vs. 0.710)^32^, but lower than the concentration of AFP in gravidas as measured by liquid-phase combination assay. Yamamoto found that the AUCs of AFP MoM (0.750), AFP-L3% (0.868), L3 MoM (0.949), and L3 MoM/AFP MoM (0.946), respectively, as determined by liquid-phase binding assay^35^. This revealed that different detection methods may produce different diagnostic values. However, the AUC for trisomy 21 as predicted by AFP-L2 and AFP-L3 in the present study showed a tendency to be lower than that predicted in our preliminary study (0.891, 0.824)^19^. The apparent discrepancy between the former and latter may have been due to our subsequent expansion of the number of control cases, or it may have related to our modeling after calibrating for gestational age and weight in the current study.

The AFP levels in the control group were higher than gravidas with trisomy 21, 18 fetuses (all *P* < 0.001), while the levels of AFP-L2 and AFP-L3 were higher than those in the control subjects (all *P* < 0.001). The maternal serum AFP-L2 and AFP-L3 levels with trisomy 21, 18 fetuses were also higher than in the control subjects, which was opposite to the diminution in AFP levels. At present, the mechanism (s) underlying this discrepant phenomenon is unclear.

Yamamoto et al. posited that the placental transfer of the AFP-L3 component in women carrying a fetus with trisomy 21 may be relatively high, which might be one of the causes for the elevated serum AFP-L3 levels in these pregnant women^36^. In addition, Yamamoto^37^ did not detect a correlation between maternal serum AFP-L3 and AFP MoMs (r = 0.006) and did not observe a significant correlation between serum AFP level and AFP-L3 percentage. No significant correlation was observed between serum AFP level and AFP-l3 percentage (r = 0.160) by Khien et al.^38^.

In our last study, the MoM values of AFP-L2 and AFP-L3 were calculated by indirect simulation based on the AFP MoM values of 21,656 middle-pregnancy maternal serum samples in our laboratory^24^. So we used the 569 maternal serum samples in this study to build the risk model construction of AFP-L2 and AFP-L3. These results all disproved any correlation between the values of AFP-L2/AFP-L3 and AFP. In support of this, our data also suggested that the combination of AFP-L2 and AFP-L3 was better than that for AFP, AFP-L3, and AFP-L2 as single indicators in predicting fetal trisomies 21, 18.

For trisomy 21, the results revealed that the substitution of AFP-L2 for AFP improved the IDI and NRI by 9.56% and 26.50%, respectively, that AFP-L3 substitution for AFP augmented the IDI and NRI by 12.34% and 26.70%, respectively. For trisomy 18, AFP-L2 replacement of AFP decreased the IDI and NRI by 8.12% and 13.84%, and that AFP-L3 replacement of AFP lowered the IDI and NRI by 1.52% and 8.54% in Table 4, respectively.

NRI is an approach that involves classifying patients into risk categories and determining how does the new model reclassify patients into risk categories compared to previous models^39^. The IDI calculation is another way of assessing reclassification that does not rely on a pre-specified risk category but represents a continuous measure^40,41^. The addition of AFP-L2 and AFP-L3 to the traditional model improved the ability to predict T21 and T18 fetuses assessed by ROC and to reclassify subjects into different risk categories by NRI and IDI. It shows that AFP-L2 and AFP-L3 can effectively increase the accuracy of previous studies.

The data in Table 4 also suggested that the utility of AFP-L3 and AFP-L2 alone in predicting trisomy 21 was better than that with AFP, while for trisomy 18, the ranked sequence was AFP-L3 > AFP > AFP-L2. If combined with DR, FPR, and +LR evaluation indicators, Model I was optimal, followed by Model F and Model E.

When both false-positive and false-negative results are inevitable, it is incumbent upon us to find a way to maximize net benefit, which is a clinical utility problem. Therefore, we introduced DCA to evaluate the predictive effects of different models for trisomy 21 and 18. It is generally postulated that the threshold probability range of the abscissa in risk threshold is 0–1. However, if a specific situation is consistent in clinical practice and the threshold probability reaches a certain value (e. g., 40%), intervention measures must be undertaken^42^ and, therefore, a risk threshold > 0.4 is of little significance. Figure 3 shows depicts the ranked order as Model A > Model G > Model I. The top models are Model B, Model E, Model I, and Model D using the DCA prediction for trisomy 18.

We note that these results were different from previous AUC, IDI, and NRI evaluation indices, which might be due to the small number of cases of trisomy 18 in the current study^18,19^.

## Conclusion

In summary, the prediction using AFP-L2 and AFP-L3 for trisomy 21 and trisomy 18 fetuses in maternal serum exhibited high sensitivity and specificity, thus providing a favorable marker for screening these trisomies. We demonstrated that the combined screening results were better than using single markers, and that the combined predictive efficiency by Model I achieved optimal results.

## Supplementary Information

Supplementary Information 1.

Supplementary Information 2.

## Data Availability

All data generated or analysed during this study are included in this published article (and its Supplementary Information files).

## Abbreviations

MoM: Multiple of the median
AFP: Alpha-fetoprotein variants
DS: Down syndrome
NTDs: Neural tube defects
free β-hCG: Free human chorionic gonadotropin
DR: Detection rate
PAPP-A: Pregnancy-associated plasma protein A
GA: Gestational age
+LR: Positive likelihood ratio
−LR: Negative likelihood ratio
IDI: Integrated discrimination improvement
NRI: Net reclassification improvement indicators
AUC: Area under the curve
DCA: Decision-curve analysis
H-L test: Hosmer–lemeshow (H-L) test
FPR: False-positive rate
ELISA: Enzyme-linked immunosorbent assay
